# How to manage the complicated hydatid cyst of the lung? Is any special surgical procedure more preferred?

**DOI:** 10.1186/1749-8090-8-S1-P147

**Published:** 2013-09-11

**Authors:** F Caushi, G Qirjako, I Bani, A Hatibi, I Skenduli, E Shima, A Menzelxhiu

**Affiliations:** 1 Department of Thoracic Surgery, University Hospital of Lung Diseases, Tirana, Albania; 2 Department of Statistics, University of Medicine, Tirana, Albania; 3 Department of Pathology, Regional Hospital, Lushnje, Albania; 4 Department of I.C.U., University Hospital of Lung Diseases, Tirana, Albania

## Background

Echinococcosis is a biological, medical, economical and social concern of great importance for countries of Mediterranean area. The treatment of this disease has been successful including complicated cysts or not. Generally at the time of diagnosis simple cysts, or healthy ones which are intact, make 65% of the cysts, meanwhile the complicated cysts make 35% of them.

## Methods

The medical data’s for 181 patients of pulmonary hydatidosis treated in our clinic were retrospectively investigated. 145 of them (80%) were treated surgically. 93 of them (51, 4%) were male and 88 (48, 6%) were females with a mean age 40 years (range 12-80 years). All the data were analyzed referring to Anova and χ² test.

## Results

31.1% of cases were intact cysts and 68, 9% were complicated cysts. 34, 8% of intervened cases had complications, mainly air leakage (>7 days) but there were not fatal cases. The cases with preoperative complicated cysts had postoperative complications 1.6 times more than for cases with simple cysts but there is no statistical significance. The mean number of the hospitalized days for complicated cysts was (18.46±7.3) and the mean numbers of hospitalized days for intact cysts (14.7+5.4) that has a statistical significance. The major part of patients with complicated cysts (60%) had undergone an On Plat procedure, but there is no statistical significance between surgical method applied for preoperative complicated cysts concerning to postoperative complications.

## Conclusions

Unlike other studies, in the major part of our cases the hydatid cysts were complicated. Although we didn’t find any statistical significance connection between preoperative complicated cysts and postoperative complications, we concluded that complicated hydatid cysts prolong the hospitalized days of the patients, so it’s better to treat them surgically at early stages. We also concluded that there is not a statistical preferred surgical technique for preoperative complicated cysts.

